# Supplementary material for: Cellular uptake of allicin in the hCMEC/D3 human brain endothelial cells: exploring blood-brain barrier penetration in an in vitro model
Source: PeerJ. 2024 Jul 17;12:e17742. doi: 10.7717/peerj.17742 (PMC11260074; doi:10.7717/peerj.17742)
Supplement: Data S1 [file peerj-12-17742-s001.docx]

**Determination of allicin in the *in vitro* BBB model by HPLC analysis**

Chromatogram of Hank’s Balanced Salt Solution (HBSS) or baseline


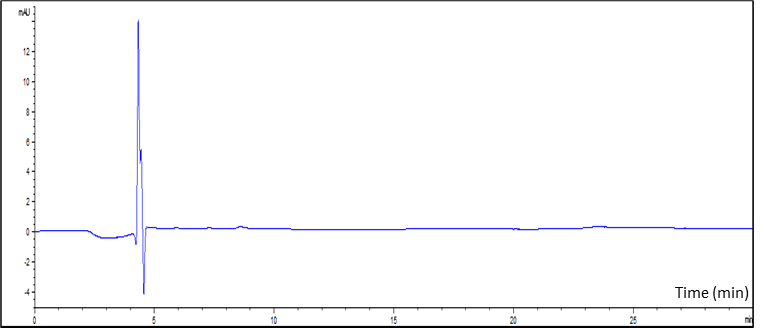


Chromatogram of allicin standard curve at 0.5 µg/ml


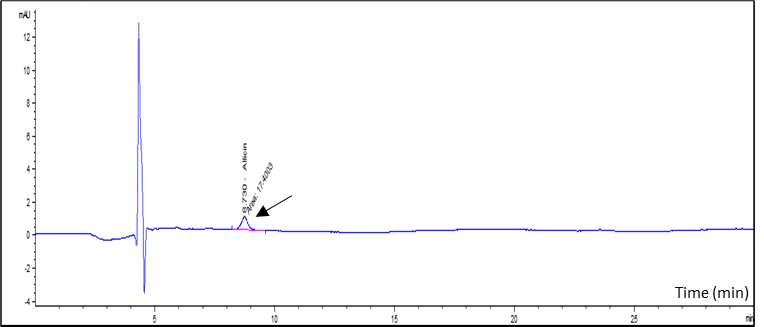


Chromatogram of allicin standard curve at 1 µg/ml


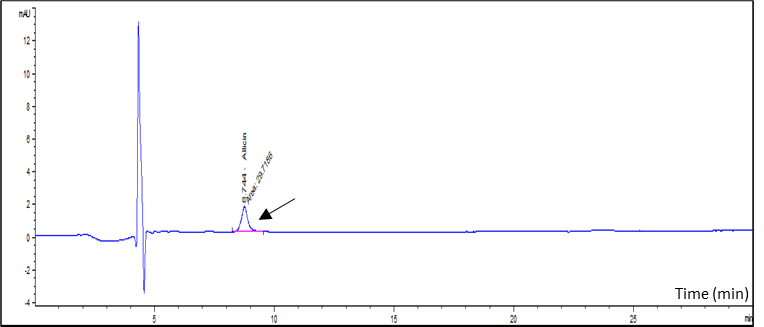


Chromatogram of allicin standard curve at 1.5 µg/ml


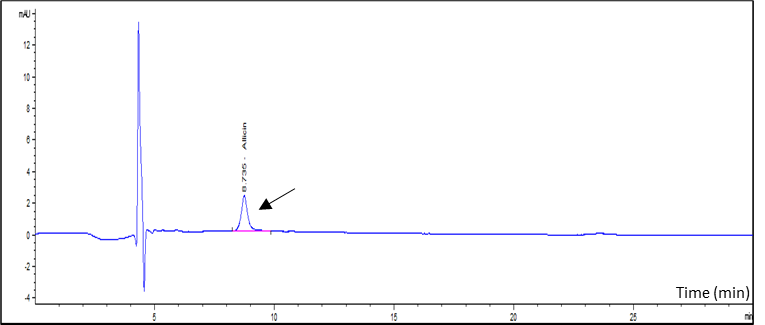


Chromatogram of allicin standard curve at 2 µg/ml


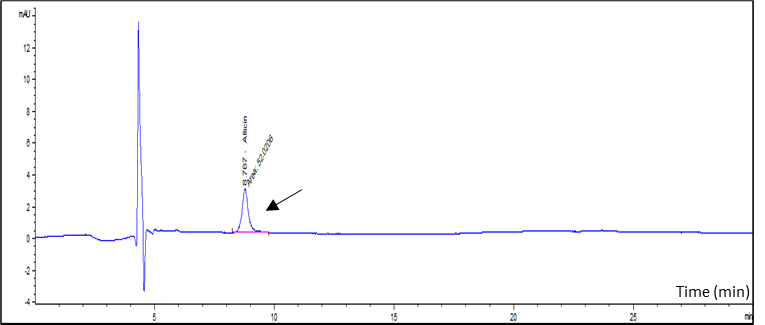


Chromatogram of allicin standard curve at 3 µg/ml


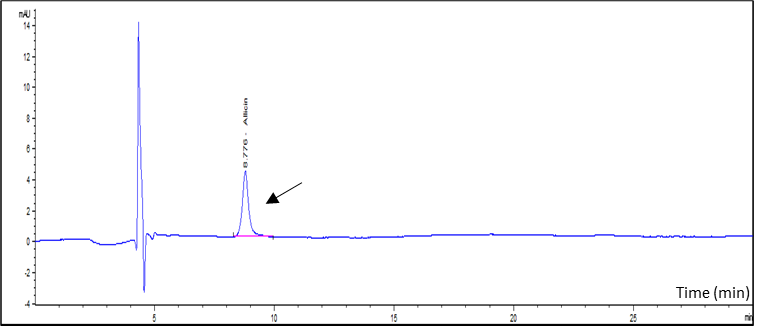


Chromatogram of allicin standard curve at 4 µg/ml


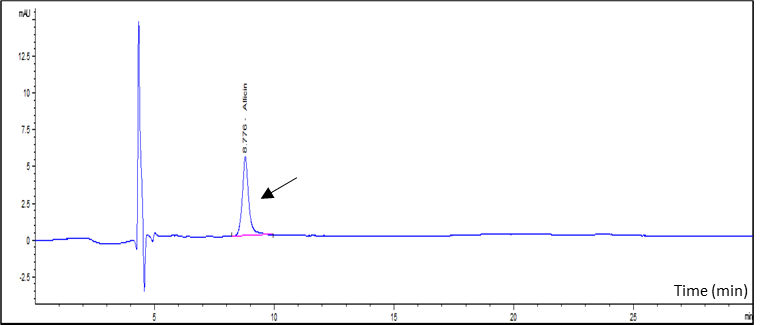


Chromatogram of allicin standard curve at 5 µg/ml


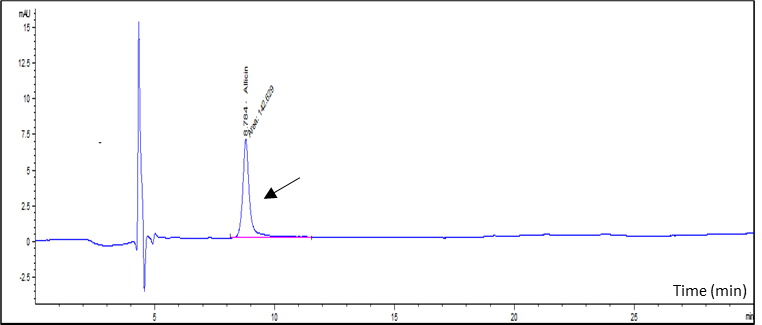


| Retention time (min) | Allicin concentration (µg/ml) | Area peak |
| --- | --- | --- |
| 8.784 | 0.5 | 17.40026 |
|  | 1 | 29.71859 |
|  | 1.5 | 43.66598 |
|  | 2 | 52.02061 |
|  | 3 | 80.93719 |
|  | 4 | 102.21091 |
|  | 5 | 142.62917 |


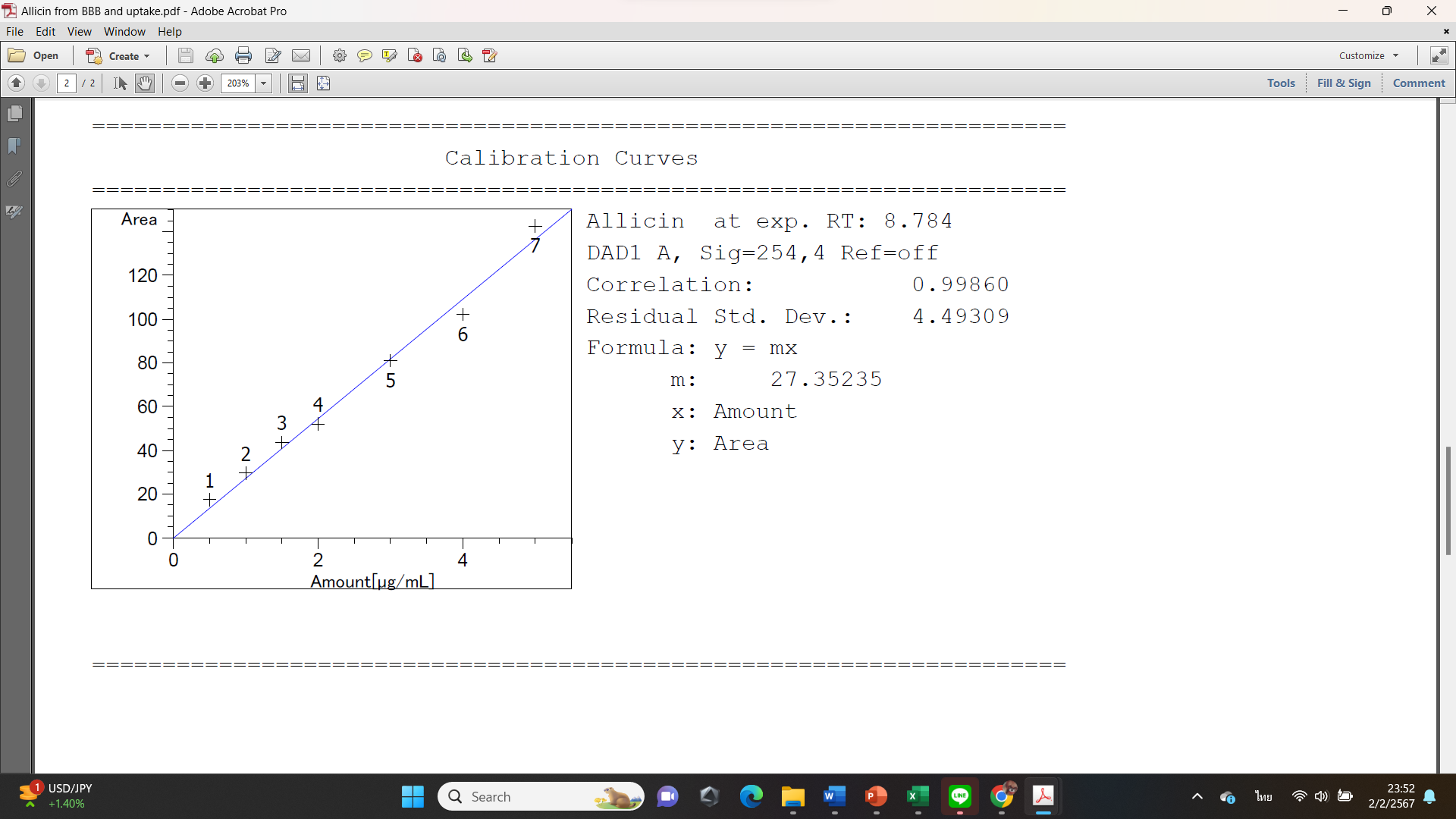


The *in vitro* BBB model tested with allicin 0.5 µg/ml

Chromatogram of the apical chamber (AP)


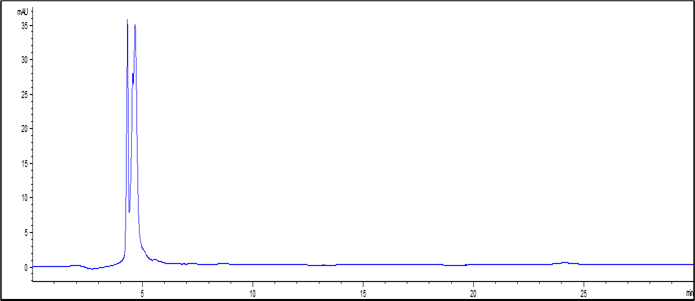


Chromatogram of the basolateral chamber (BL)


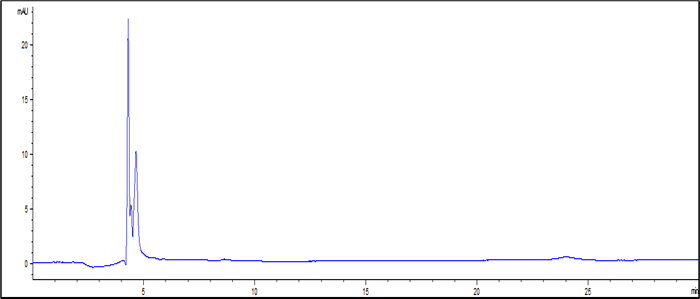


The *in vitro* BBB model tested with allicin 1 µg/ml

Chromatogram of the apical chamber (AP)


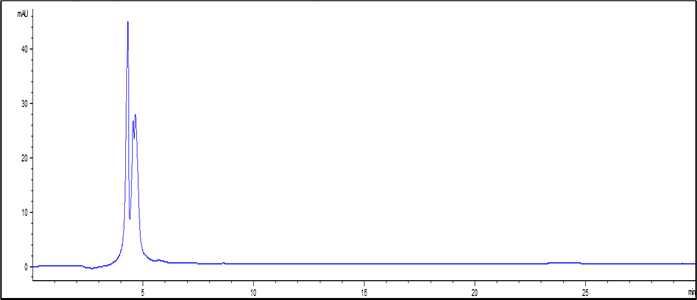


Chromatogram of the basolateral chamber (BL)


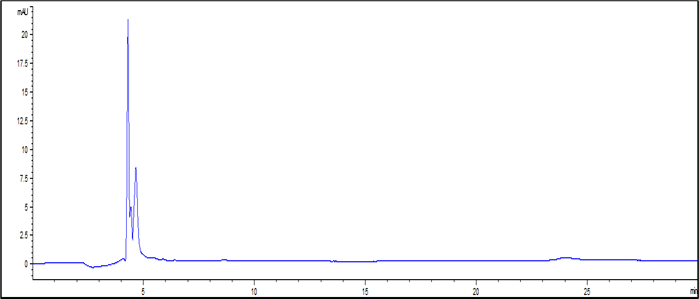


The *in vitro* BBB model tested with allicin 2 µg/ml

Chromatogram of the apical chamber (AP)


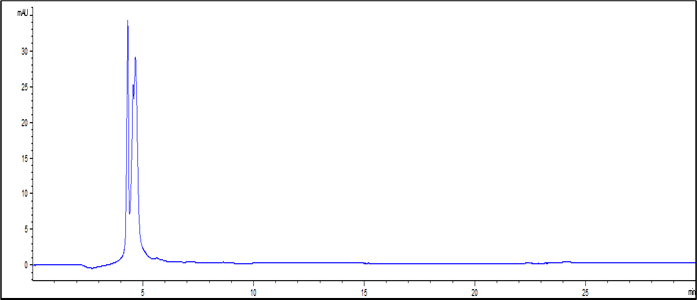


Chromatogram of the basolateral chamber (BL)


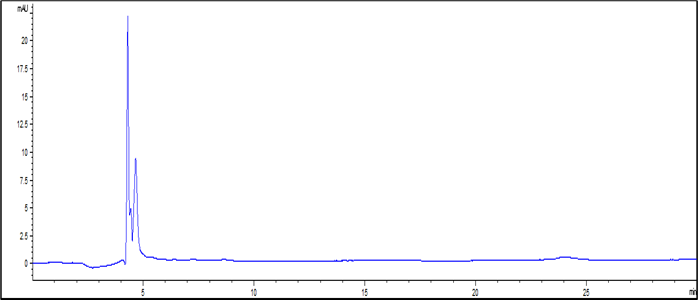


The *in vitro* BBB model tested with allicin 5 µg/ml

Chromatogram of the apical chamber (AP)


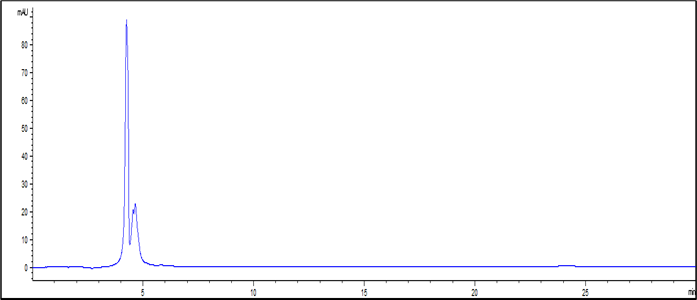


Chromatogram of the basolateral chamber (BL)


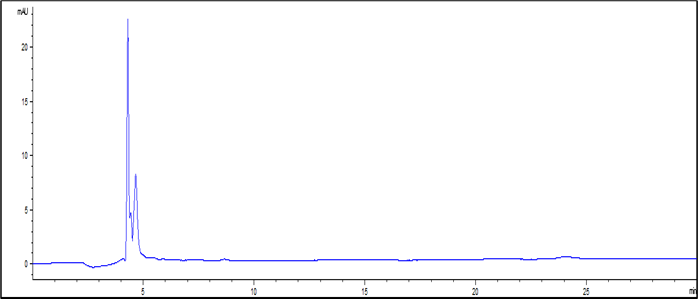


The cell-free control insert tested with allicin 5 µg/ml

Chromatogram of the apical chamber (AP)


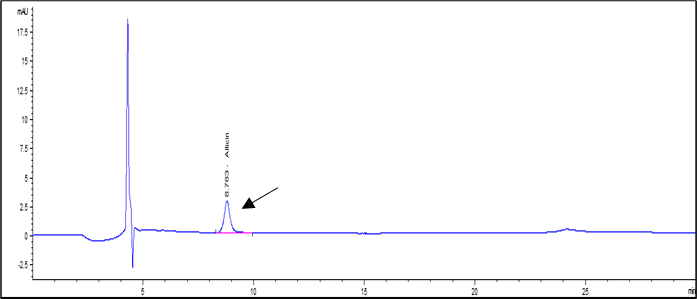


Chromatogram of the basolateral chamber (BL)


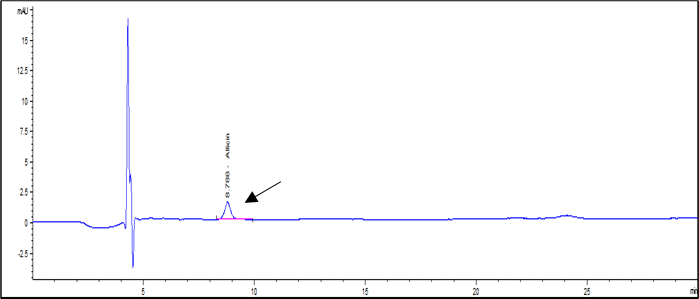


Allicin concentration of AP and BL samples of the cell-free control insert

| Sample | Allicin concentration (µg/ml) |
| --- | --- |
| AP | 1.97 |
| BL | 1.09 |

***In Silico* ADME and Toxicity Profiling Predictions**

**Molecule Depiction**


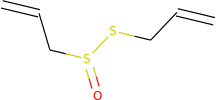


SMILES

**Molecule properties:**

| **Descriptor** | **Value** |
| --- | --- |
| Molecular Weight | 162.279 |
| LogP | 1.7553 |
| #Rotatable Bonds | 5 |
| #Acceptors | 2 |
| #Donors | 0 |
| Surface Area | 62.082 |

| **Property** | **Model Name** | **Predicted Value** | **Unit** |
| --- | --- | --- | --- |
| **Absorption** | Water solubility | **-1.726** | Numeric (log mol/L) |
| **Absorption** | Caco2 permeability | **1.313** | Numeric (log Papp in 10^-6^ cm/s) |
| **Absorption** | Intestinal absorption (human) | **96.468** | Numeric (% Absorbed) |
| **Absorption** | Skin Permeability | **-1.869** | Numeric (log Kp) |
| **Absorption** | P-glycoprotein substrate | **No** | Categorical (Yes/No) |
| **Absorption** | P-glycoprotein I inhibitor | **No** | Categorical (Yes/No) |
| **Absorption** | P-glycoprotein II inhibitor | **No** | Categorical (Yes/No) |
| **Distribution** | VDss (human) | **-0.041** | Numeric (log L/kg) |
| **Distribution** | Fraction unbound (human) | **0.575** | Numeric (Fu) |
| **Distribution** | BBB permeability | **0.51** | Numeric (log BB) |
| **Distribution** | CNS permeability | **-2.312** | Numeric (log PS) |
| **Metabolism** | CYP2D6 substrate | **No** | Categorical (Yes/No) |
| **Metabolism** | CYP3A4 substrate | **No** | Categorical (Yes/No) |
| **Metabolism** | CYP1A2 inhibitior | **No** | Categorical (Yes/No) |
| **Metabolism** | CYP2C19 inhibitior | **No** | Categorical (Yes/No) |
| **Metabolism** | CYP2C9 inhibitior | **No** | Categorical (Yes/No) |
| **Metabolism** | CYP2D6 inhibitior | **No** | Categorical (Yes/No) |
| **Metabolism** | CYP3A4 inhibitior | **No** | Categorical (Yes/No) |
| **Excretion** | Total Clearance | **0.714** | Numeric (log ml/min/kg) |
| **Excretion** | Renal OCT2 substrate | **No** | Categorical (Yes/No) |
| **Toxicity** | AMES toxicity | **No** | Categorical (Yes/No) |
| **Toxicity** | Max. tolerated dose (human) | **0.708** | Numeric (log mg/kg/day) |
| **Toxicity** | hERG I inhibitor | **No** | Categorical (Yes/No) |
| **Toxicity** | hERG II inhibitor | **No** | Categorical (Yes/No) |
| **Toxicity** | Oral Rat Acute Toxicity (LD50) | **2.344** | Numeric (mol/kg) |
| **Toxicity** | Oral Rat Chronic Toxicity (LOAEL) | **1.431** | Numeric (log mg/kg_bw/day) |
| **Toxicity** | Hepatotoxicity | **No** | Categorical (Yes/No) |
| **Toxicity** | Skin Sensitisation | **Yes** | Categorical (Yes/No) |
| **Toxicity** | *T.Pyriformis* toxicity | **0.898** | Numeric (log ug/L) |
| **Toxicity** | Minnow toxicity | **1.205** | Numeric (log mM) |

**Molecule Depiction**


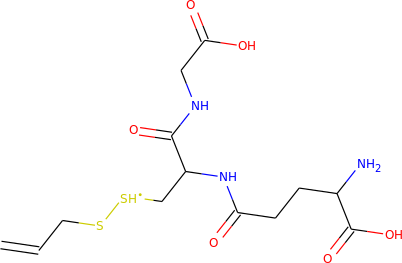


SMILES

**Molecule properties:**

| **Descriptor** | **Value** |
| --- | --- |
| Molecular Weight | 380.468 |
| LogP | -0.8362 |
| #Rotatable Bonds | 13 |
| #Acceptors | 6 |
| #Donors | 6 |
| Surface Area | 146.749 |

| **Property** | **Model Name** | **Predicted Value** | **Unit** |
| --- | --- | --- | --- |
| **Absorption** | Water solubility | **-2.53** | Numeric (log mol/L) |
| **Absorption** | Caco2 permeability | **-0.401** | Numeric (log Papp in 10^-6^ cm/s) |
| **Absorption** | Intestinal absorption (human) | **0** | Numeric (% Absorbed) |
| **Absorption** | Skin Permeability | **-2.735** | Numeric (log Kp) |
| **Absorption** | P-glycoprotein substrate | **Yes** | Categorical (Yes/No) |
| **Absorption** | P-glycoprotein I inhibitor | **No** | Categorical (Yes/No) |
| **Absorption** | P-glycoprotein II inhibitor | **No** | Categorical (Yes/No) |
| **Distribution** | VDss (human) | **-1.321** | Numeric (log L/kg) |
| **Distribution** | Fraction unbound (human) | **0.727** | Numeric (Fu) |
| **Distribution** | BBB permeability | **-1.244** | Numeric (log BB) |
| **Distribution** | CNS permeability | **-3.475** | Numeric (log PS) |
| **Metabolism** | CYP2D6 substrate | **No** | Categorical (Yes/No) |
| **Metabolism** | CYP3A4 substrate | **No** | Categorical (Yes/No) |
| **Metabolism** | CYP1A2 inhibitior | **No** | Categorical (Yes/No) |
| **Metabolism** | CYP2C19 inhibitior | **No** | Categorical (Yes/No) |
| **Metabolism** | CYP2C9 inhibitior | **No** | Categorical (Yes/No) |
| **Metabolism** | CYP2D6 inhibitior | **No** | Categorical (Yes/No) |
| **Metabolism** | CYP3A4 inhibitior | **No** | Categorical (Yes/No) |
| **Excretion** | Total Clearance | **0.251** | Numeric (log ml/min/kg) |
| **Excretion** | Renal OCT2 substrate | **No** | Categorical (Yes/No) |
| **Toxicity** | AMES toxicity | **No** | Categorical (Yes/No) |
| **Toxicity** | Max. tolerated dose (human) | **1.006** | Numeric (log mg/kg/day) |
| **Toxicity** | hERG I inhibitor | **No** | Categorical (Yes/No) |
| **Toxicity** | hERG II inhibitor | **No** | Categorical (Yes/No) |
| **Toxicity** | Oral Rat Acute Toxicity (LD50) | **1.66** | Numeric (mol/kg) |
| **Toxicity** | Oral Rat Chronic Toxicity (LOAEL) | **2.71** | Numeric (log mg/kg_bw/day) |
| **Toxicity** | Hepatotoxicity | **Yes** | Categorical (Yes/No) |
| **Toxicity** | Skin Sensitisation | **No** | Categorical (Yes/No) |
| **Toxicity** | *T.Pyriformis* toxicity | **0.285** | Numeric (log ug/L) |
| **Toxicity** | Minnow toxicity | **4.089** | Numeric (log mM) |
