# Supplementary material for: Cellular uptake of allicin in the hCMEC/D3 human brain endothelial cells: exploring blood-brain barrier penetration in an in vitro model
Source: PeerJ. 2024 Jul 17;12:e17742. doi: 10.7717/peerj.17742 (PMC11260074; doi:10.7717/peerj.17742)

Supplemental information

Figure 1 Determination of allicin in the *in vitro* BBB model by HPLC analysis

Chromatogram of Hank’s Balanced Salt Solution (HBSS) or baseline


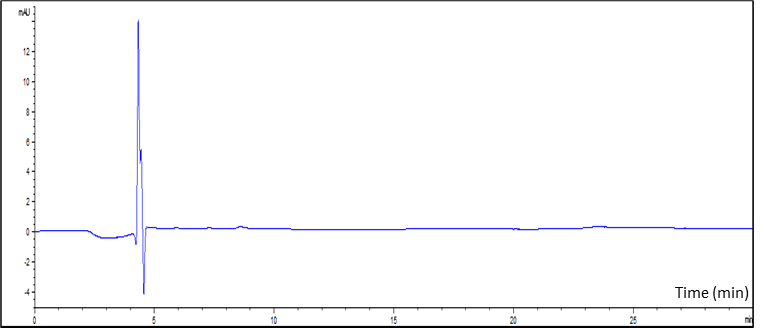


Chromatogram of allicin standard curve at 0.5 µg/ml


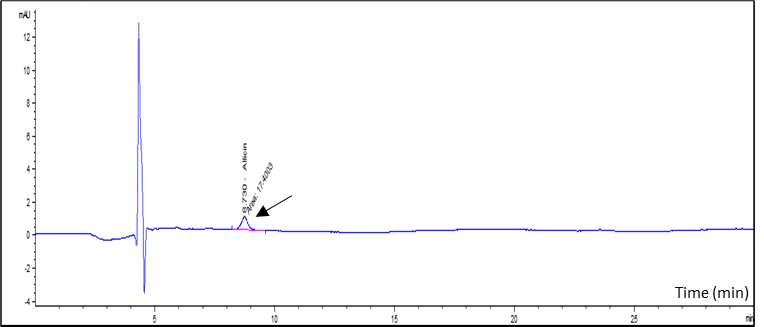


Chromatogram of allicin standard curve at 1 µg/ml


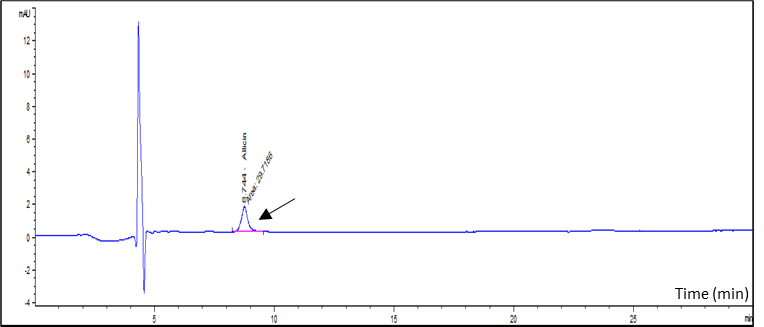


Chromatogram of allicin standard curve at 1.5 µg/ml


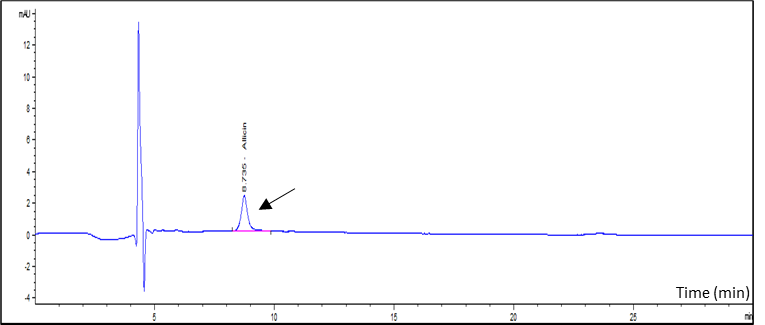


Chromatogram of allicin standard curve at 2 µg/ml


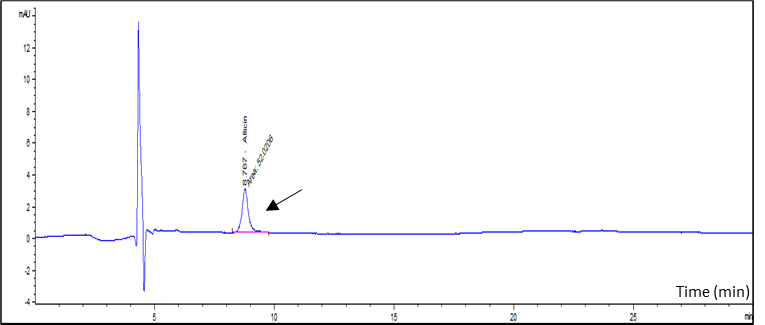


Chromatogram of allicin standard curve at 3 µg/ml


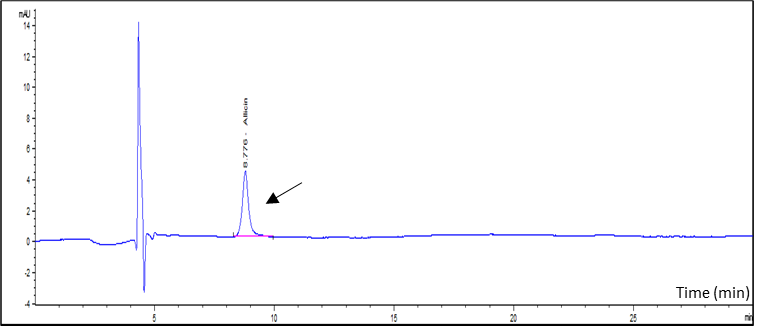


Chromatogram of allicin standard curve at 4 µg/ml


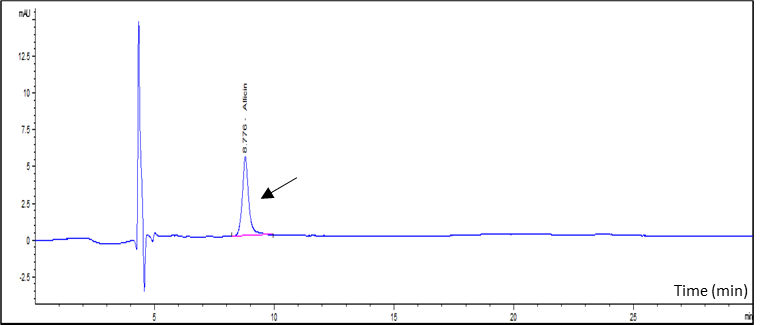


Chromatogram of allicin standard curve at 5 µg/ml


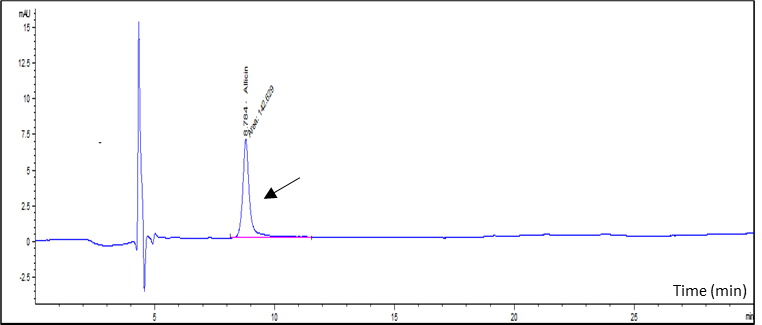

Supplement: Supplemental Information 3 [file peerj-12-17742-s003.docx]
